# Supplementary material for: Mobocertinib (TAK-788) in EGFR Exon 20 Insertion+ Metastatic NSCLC: Patient-Reported Outcomes from EXCLAIM Extension Cohort
Source: J Clin Med. 2022 Dec 23;12(1):112. doi: 10.3390/jcm12010112 (PMC9821270; doi:10.3390/jcm12010112)
Supplement: Supplementary file 1 [file jcm-12-00112-s001.zip › jcm-2078452-supplementary.pdf]

Table S1. **Summary of Compliance With PRO Questionnaires at Visit Level**

Timepoint	Forms Received	Forms Expected	Compliance Rate
**EORTC QLQ-C30**			
Baseline	90	90	100.0%
C2D1	86	88	97.7%
C3D1	83	83	100.0%
C4D1	77	78	98.7%
C5D1	68	68	100.0%
C6D1	61	62	98.4%
C7D1	54	56	96.4%
C8D1	47	50	94.0%
C9D1	38	39	97.4%
C10D1	34	36	94.4%
C11D1	30	31	96.8%
C12D1	30	31	96.8%
C13D1	25	25	100.0%
C14D1	25	25	100.0%
C15D1	25	25	100.0%
C16D1	1	1	100.0%
C17D1	1	1	100.0%
C18D1	22	23	95.7%
EOT	43	67	64.2%
30D ALD	26	47	55.3%
**EORTC QLQ-LC13**			
Baseline	90	90	100.0%
C2D1	86	88	97.7%
C3D1	83	83	100.0%
C4D1	77	78	98.7%
C5D1	68	68	100.0%
C6D1	61	62	98.4%
C7D1	54	56	96.4%
C8D1	47	50	94.0%
C9D1	38	39	97.4%
C10D1	34	36	94.4%
C11D1	30	31	96.8%
C12D1	30	31	96.8%
C13D1	25	25	100.0%
C14D1	24	25	96.0%

Timepoint	Forms Received	Forms Expected	Compliance Rate
C15D1	25	25	100.0%
C16D1	1	1	100.0%
C17D1	1	1	100.0%
C18D1	22	23	95.7%
EOT	44	67	65.7%
30D ALD	25	47	53.2%
**EQ-5D-5L**			
Baseline	90	90	100.0%
C2D1	85	88	96.6%
C3D1	83	83	100.0%
C4D1	77	78	98.7%
C5D1	68	68	100.0%
C6D1	61	62	98.4%
C7D1	54	56	96.4%
C8D1	47	50	94.0%
C9D1	38	39	97.4%
C10D1	34	36	94.4%
C11D1	30	31	96.8%
C12D1	30	31	96.8%
C13D1	25	25	100.0%
C14D1	25	25	100.0%
C15D1	25	25	100.0%
C16D1	1	1	100.0%
C17D1	1	1	100.0%
C18D1	22	23	95.7%
EOT	43	67	64.2%
30D ALD	26	47	55.3%
**PRO-CTCAE**			
Baseline	80	80	100.0%
C2D1	85	88	96.6%
C3D1	83	83	100.0%
C4D1	77	78	98.7%
C5D1	68	68	100.0%
C6D1	61	62	98.4%
C7D1	54	56	96.4%
C8D1	47	50	94.0%
C9D1	38	39	97.4%
C10D1	34	36	94.4%

Timepoint	Forms Received	Forms Expected	Compliance Rate
C11D1	30	31	96.8%
C12D1	30	31	96.8%
C13D1	25	25	100.0%
C14D1	24	25	96.0%
C15D1	25	25	100.0%
C16D1	1	1	100.0%
C17D1	1	1	100.0%
C18D1	22	23	95.7%
EOT	42	67	62.7%
30D ALD	25	47	53.2%

30D ALD, indicates 30 days after last dose; C, cycle; D, day; EORTC QLQ-C30, European Organisation for Research and Treatment of Cancer Core Quality-of-Life Questionnaire; EORTC QLQ-LC13, European Organisation for Research and Treatment of Cancer Core Quality-of-Life Questionnaire lung cancer module; EOT, end of treatment; EQ-5D-5L, EuroQol-5 Dimensions-5 Levels; PRO-CTCAE, Patient-Reported Outcomes Version of the Common Terminology Criteria for Adverse Events
